# Undifferentiated embryonal sarcoma of the liver occurring in an adolescent: a case report with genomic analysis

**DOI:** 10.1186/s40792-022-01528-0

**Published:** 2022-09-15

**Authors:** Tomonari Shimagaki, Keishi Sugimachi, Yohei Mano, Emi Onishi, Yuki Tanaka, Rie Sugimoto, Kenichi Taguchi, Masaru Morita, Yasushi Toh

**Affiliations:** 1 Department of Hepatobiliary and Pancreatic Surgery, National Hospital Organization Kyushu Cancer Center, 3-1-1 Notame, Minami-ku, Fukuoka, 811-1395 Japan; 2 Department of Hepato-Biliary-Pancreatology, National Hospital Organization Kyushu Cancer Center, Fukuoka, 811-1395 Japan; 3 Department of Pathology, National Hospital Organization Kyushu Cancer Center, Fukuoka, 811-1395 Japan; 4 National Hospital Organization Kyushu Cancer Center, Fukuoka, 811-1395 Japan

**Keywords:** Undifferentiated embryonal sarcoma of the liver, Surgical resection, Adjuvant chemotherapy, Cancer genomic medicine

## Abstract

**Background:**

Undifferentiated embryonal sarcoma of the liver (UESL) is a rare malignant mesenchymal tumor that usually occurs in children and is rarely diagnosed in adults.

**Case presentation:**

The case was a female in her late 20s who presented with a huge liver mass found upon the examination of fever. Imaging analysis showed a well-defined mass measuring 9 cm in the largest dimension in the right posterior segment of the liver. The patient underwent right hemi-hepatectomy. Histopathological studies revealed that the circumscribed tumor was composed of a proliferation of atypical epithelioid to spindle-shaped cells with pleomorphic nuclei arranged in haphazard pattern. Histopathological features observed in immunohistochemical analyses confirmed a final diagnosis of UESL. Genome analysis using FoundationOne CDx revealed 11 somatic mutations including *TP53* (R196*) and *STK11* (F354L). Adjuvant chemotherapy with ifosfamide and etoposide was performed, and the case has been followed up without recurrence for 1 year after hepatectomy.

**Conclusions:**

A UESL should be considered in the differential diagnosis of large and well-defined solid liver lesions. Although the prognosis of UESL is extremely unfavorable, aggressive surgical resection with adjuvant chemotherapy and genomic analysis may be helpful for ensuring long-term survival.

## Background

Undifferentiated embryonal sarcoma of the liver (UESL) is an extremely rare entity in adulthood, with fewer than 60 cases reported in the literature [1–3]. Embryonal sarcoma more typically occurs in children, with a peak incidence between the ages of 6 and 10 years with no sex predilection [4]. Embryonal sarcoma represents the third most common primary pediatric liver tumor after hepatoblastoma and hepatocellular carcinoma [5]. The behavior of UESL is generally highly aggressive in children and is considered to be the same in adults [6]. However, preoperative diagnosis of UESL is rarely accurate for adults. Surgical resection offers the chance of possible cure and should be considered in all cases [5]. We herein reported a case of UESL in an adult patient treated by radical surgery and discuss the features of UESL and outcomes considering adjuvant chemotherapy and the results of genomic analysis.

## Case presentation

A woman in her late 20s was admitted because of a huge liver mass detected during evaluation for fever and abdominal pain. She initially came to our hospital with fever, intermittent right quadrant abdominal pain for 1 week and a palpable abdominal mass. The patient had a medical history of extensive resection and postoperative adjuvant chemotherapy for osteosarcoma of the left femur 15 years previously. Serum levels of tumor markers (carcinoembryonic antigen (CEA), CA19-9, alpha-fetoprotein (AFP), and des-gamma-carboxy prothrombin were all within normal limits and she was negative for hepatic viral markers (HBsAg and anti-HCV). The laboratory data revealed no specific findings except for elevated C-reactive protein (8.56 mg/dL).

**Fig. 1:**
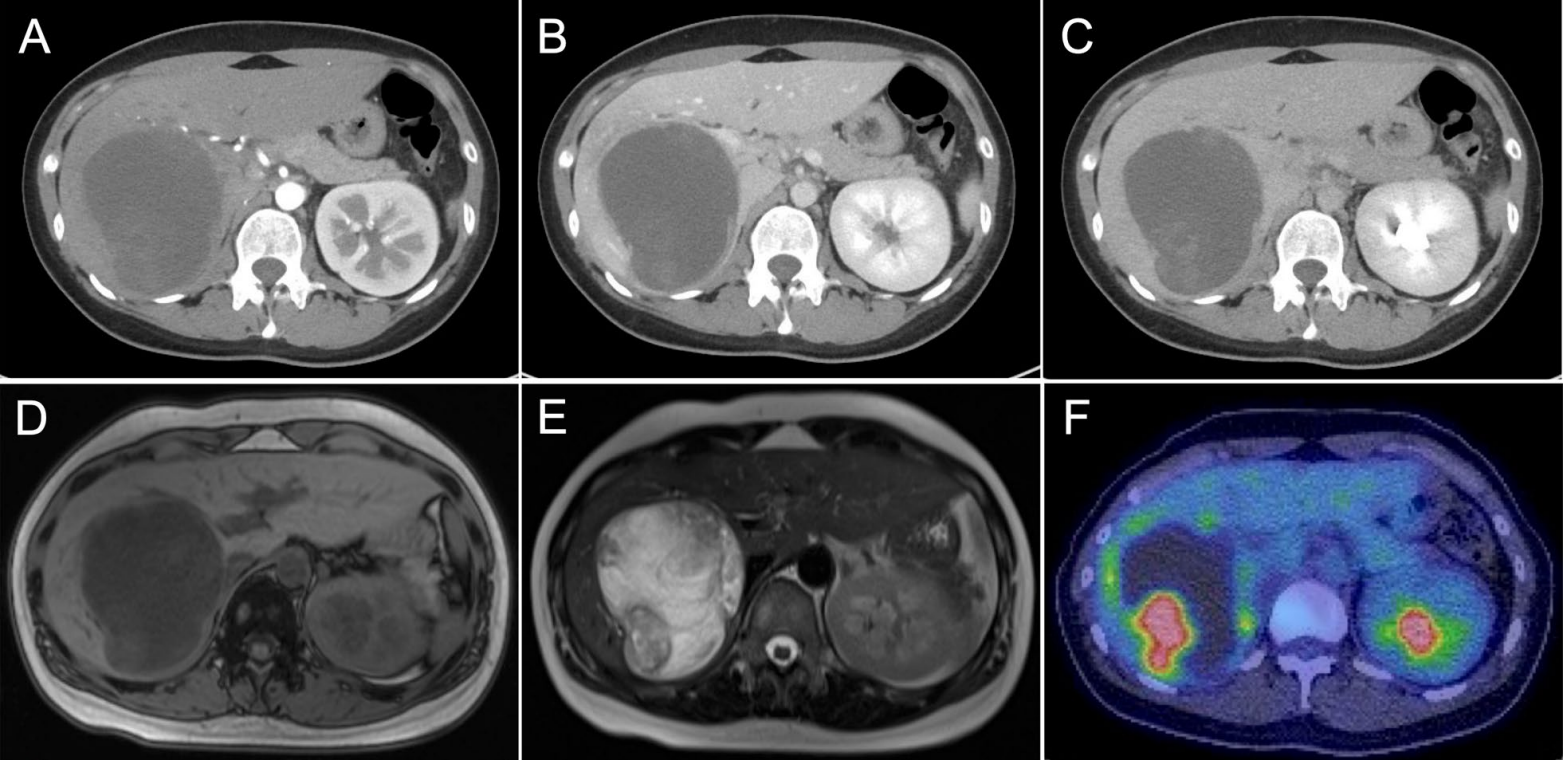
Preoperative imaging findings. CT imaging demonstrating the hepatic tumor (axial section): **A** arterial phase, **B** portal phase, and **C** equilibrium phase. MRI imaging demonstrating the hepatic tumor: **D** T1-weighted and **E** T2-weighted images. **F** Positron emission tomography (PET) imaging

Computed tomography showed a well-defined mass measuring 9 cm in the largest dimension in the right posterior segment of the liver. The tumor was slightly enhanced, but a major part was hypo-vasculated (Fig. 1A–C). Magnetic resonance imaging showed a mixed intensity of high and low signals. The cysts had low signal intensity on T1-weighted images and high signal intensity on T2-weighted images, suggesting a high water content. Furthermore, areas with a partially high signal intensity on T1-weighted images and a low signal intensity on T2-weighted images were suggestive of intratumoral hemorrhage (Fig. 1D, E). Positron emission tomography showed the uptake of 2-(fluorine-18)-fluoro-2-deoxy-d-glucose in part of the solid components of the cyst (SUV-max 11.11; Fig. 1F).

**Fig. 2:**
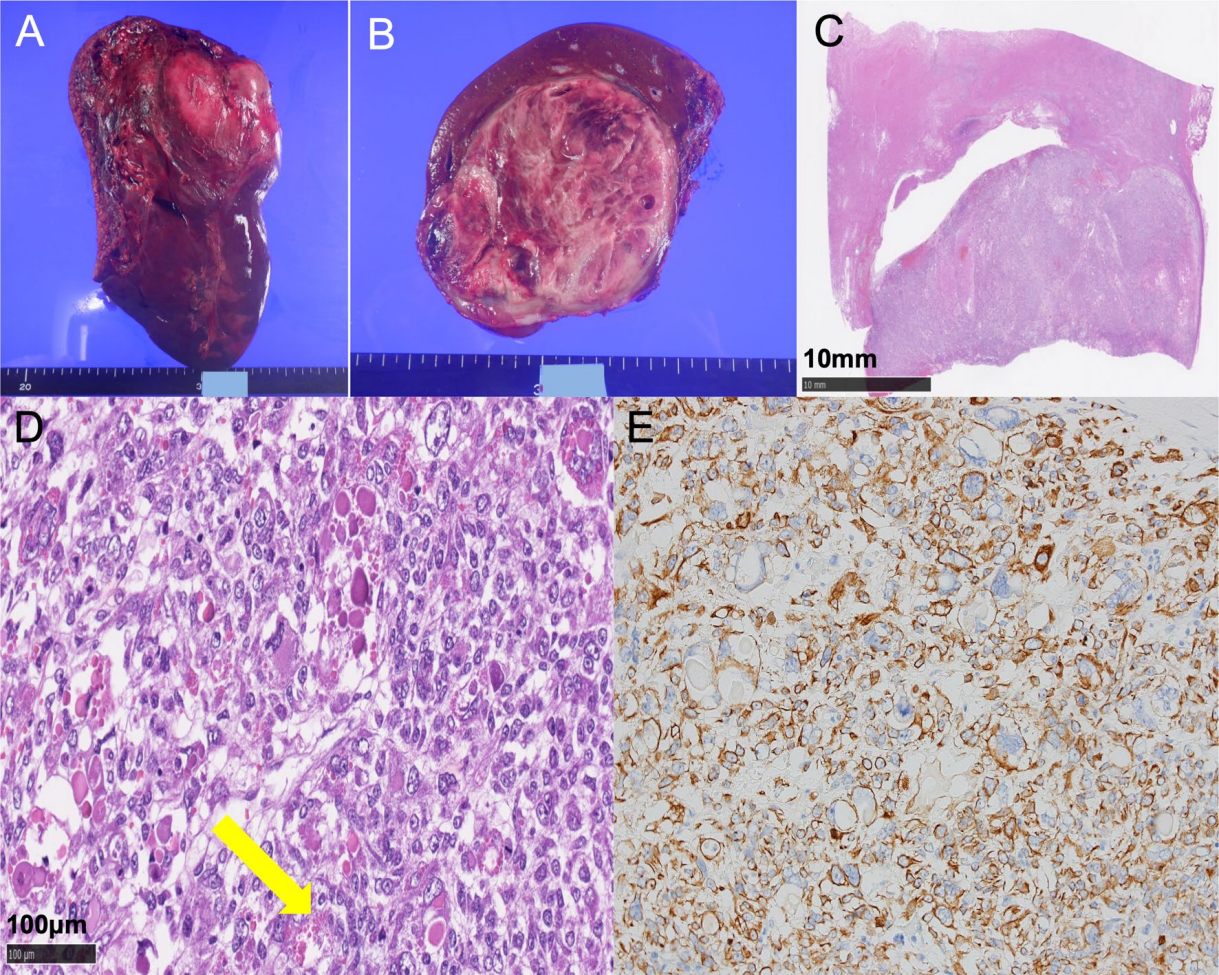
Postoperative specimen findings. **A**, **B** Macroscopic findings of the surgical specimen. **C**, **D** Microscopic findings. **C** HE: × 10 (bar, 10 mm) and **D** HE: × 200 (bar, 100 μm). Cells with granular vesicles positive for PAS were scattered in the cytoplasm (yellow arrow). **E** Immunohistochemical analyses. AE1/AE3: × 200

A liver needle biopsy was performed due to the possibility of recurrence of osteosarcoma. Histopathological studies showed a proliferation of malignant pleomorphic atypical cells associated with necrosis, and the tumor cells were positive for epithelial marker (AE1/AE3 and CAM5.2) by immunohistochemical analyses. The patient was diagnosed with an unspecified malignant tumor, ruling out metastatic osteosarcoma or primary poorly differentiated carcinoma. Therefore, we decided to perform right hemi-hepatectomy with the aim of both treatment and definitive diagnosis. Upon laparotomy the huge tumor was observed, but extrahepatic invasion was not found. No peritoneal dissemination or ascites was observed. The right lobe was completely mobilized. Then the right hepatic artery, the right branch of portal vein, and the right hepatic duct were divided individually. The hepatic parenchyma was divided along the Cantlie line, and the right hepatic vein was divided. The right hemi-hepatectomy was performed with an uneventful course. The operation time was 6 h 34 min, and the operative blood loss was 340 ml. The resected specimen showed a heterogenic tumor measuring 9.0 × 6.5 × 4.5 cm. The cut surface of the specimen revealed a multilocular mass with the various components of hemorrhage, necrosis, and a mucinous substance (Fig. 2A, B).

Histopathological studies revealed that the circumscribed tumor was composed of a proliferation of atypical epithelioid to spindle-shaped cells with pleomorphic nuclei arranged in haphazard pattern, accompanied by massive necrosis and hemorrhage (Fig. 2C). Cells with granular vesicles positive for periodic acid–Schiff (PAS) were scattered in the cytoplasm (Fig. 2D). Immunohistochemical analyses showed that the tumor cells were positive for pan cytokeratin (AE1/AE3) (Fig. 2E), CAM5.2, glypican 3, vimentin, and desmin, and were negative for CD34, ETS-related gene, D2-40, S-100, human melanoma black 45, melan A, and smooth muscle actin (SMA). The MIB1 labeling index was 80% in a hot spot. Therefore, a histological diagnosis of UESL was made.

We submitted the excised specimen to genomic analysis using FoundationOne CDx, which revealed 11 somatic mutations in *TP53* and *STK11*, among other genes (Table 1). One year after hepatectomy, we were able to follow up without recurrence while administering adjuvant chemotherapy with ifosfamide and etoposide. The patient received ifosfamide (1.8 g/m^2^) and etoposide (100 mg/m^2^) on days 1–5. This treatment was repeated at 28-day intervals (3 cycles). There is still no consensus on chemotherapy for UESL. Therefore, the decision of these drug was made based on a conference with medical oncologists, hepatologists, and surgeons, while referring to previous reports [5].

**Table 1:** Genomic analysis of undifferentiated embryonal sarcoma of the liver by FoundationOne CDx

Detected somatic mutation	FDA-approved therapeutic options	Clinical significance
TP53(R196*)	Doxorubicin hydrochloride	Sensitivity/response
	Abemaciclib	Resistance
STK11(F354L)	Atezolizumab	Resistance
	Nivolumab	Resistance
	Pembrolizumab	Resistance
POLE(K2223R)	–	–
AXL(P179L)	–	–
BRIP1(D546G)	–	–
IKBKE(G660E)	–	–
MEF2B(A174G)	–	–
NF2(c.114+1G>A)	–	–
PARP3(A232_E234del)	–	–
SNCAIP(T183A)	–	–
TSC1(A84T)	–	–

## Discussion

UESL is a rare primary mesenchymal tumor in children but there have been a few reports in adults as well [7, 8]. The oldest reported patient was an 86-year-old woman described by Ellis and Cotton in 1983 [9]. Because of the low incidence of UESL, especially among adults, most of the literature comprises case reports, with a limited number of small case series. These reports often present as masses with solid and cystic components. Due to the rarity of UESL in adult patients, these patients are often misdiagnosed as hepatic abscess, hemorrhage cystic tumor, or hydatic cyst, as in the current case [2]. The diagnosis of UESL is difficult because it has no specific clinical characteristics, as shown in this case. Some patients may present with various non-specific gastrointestinal symptoms, such as nausea, vomiting, abdominal pain, diarrhea, and jaundice. A large liver mass along with persistent weight loss is apparent in most adult cases. UESL is not associated with cirrhosis or chronic liver disease; thus, liver functions and tumor markers such as AFP, CEA, and CA 19-9 are normal in most cases, such as in the present case.

Macroscopically, UESL is a large, well-circumscribed mass with areas of cystic degeneration, necrosis, hemorrhage, and an occasional gelatinous appearance, and the tumor is surrounded by a fibrous pseudocapsule with direct invasion of the adjacent parenchyma, consistent with our findings. The cellular component is composed of medium-to-large spindled or stellate cells with marked nuclear pleomorphism [10, 11]. Although its pathological origin is unclear, studies have shown histiocytic, lipoblastic, myoblastic, myofibroblastic, rhabdomyoblastic, and leiomyoblastic differentiation [12]. UESLs are usually diffusely positive for vimentin and a1-antitrypsin and focally positive for cytokeratin, desmin, α-SMA, muscle-specific actin, CD68, myoglobin, neuron-specific enolase, S100, and CD34, suggesting that an embryonal sarcoma is undifferentiated [13]. PAS-positive and diastase-resistant intra- and extracytoplasmic globules may be observed in UESL [14], as was seen in our case. Histology sometimes showed sloughed-off biliary epithelial cells of preexisting bile ducts trapped between the degenerated masses and fibrous septae of the tumor.

The standard treatment of UESL includes complete resection of the tumor and combined adjuvant chemotherapy [15]. Liver transplantation may also be an option for patients with unresectable tumors [6]. Radical resection is also recommended for recurrent cases [16]. Lenze et al. reviewed reports from 1955 to 2007 and reported that the combination of surgical resection and adjuvant chemotherapy may improve prognosis compared with resection alone [17]. Although there are no standard regimens for adjuvant chemotherapy, sarcoma-directed chemotherapy such as combination of vincristine, actinomycin, ifosfamide, and doxorubicin or combination of carboplatin and etoposide has been used [18]. Drug selection for adjuvant chemotherapy was based on past reports rather than on genomic results. In this genomic results, there were no ready-to-use actionable drugs for UESL. To our knowledge, there are reports that only 6 of 16 adult patients over the age of 18 years (37.5%) who survived for more than 48 months had no recurrence after the primary surgery [17, 19]. Of the 10 patients who relapsed, eight patients (80%) presented with recurrence in the remnant liver [19]. The current patient has been disease-free for 1 year after surgery, but careful follow up is still necessary.

To date, there have been no reports of genomic comprehensive analyses of UESL cases. There have been a few studies of *TP53* mutations in some UESL cases, as well as overexpression of p53 in tumoral cells, suggesting that the p53 pathway may be involved in the carcinogenesis of UESL, similar to a number of other tumors [20, 21]. Hu et al. emphasized the inactivation of *TP53* through the loss of heterozygosity and a pathogenic mutation of the remaining allele [21]. Restoration of *TP53* function could be of interest for therapeutic strategies for UESL [21].

We considered that UESL was not the recurrence of osteosarcoma because histological types were completely different. There are three possible relationships between the present UESL and past osteosarcoma in this case: completely unrelated, secondary carcinogenesis due to chemotherapy, and genetic factors. Although the possibility of secondary carcinogenesis due to the chemotherapy for osteosarcoma could not be denied, there has been no reports that UESL occurred as a secondary cancer due to chemotherapy or radiation therapy. Taking into account that the present UESL had *TP53* mutation, the Li–Fraumeni syndrome might be possible in this case. However, we could not investigate *TP53* mutations in this case’s germline or past osteosarcoma because informed consent had not been obtained from the patient.

In the present case, genomic analysis revealed 11 somatic mutations. We identified a *STK11* mutation that has never been reported in UESL thus far. There is a report that the therapeutic effect of programmed death-(ligand) 1 (PD-1/PD-L1) inhibition was diminished in *STK11*- and *KEAP1*-mutant lung adenocarcinoma [22]. This suggests that our findings might shed new light on the clinical diagnosis and add strong evidence of a potential targeted treatment through comprehensive genome analysis and companion diagnostics.

## Conclusions

UESL should be included in the differential diagnosis of large liver masses, regardless of patient age. It is an aggressive tumor, but combined therapy of complete resection and chemotherapy may improve the prognosis. Complete resection with adjuvant chemotherapy and genomic analysis may be the next most important step for long-term survival.

## Data Availability

Not applicable.

## Abbreviations

UESL: Undifferentiated embryonal sarcoma of the liver
CT: Computed tomography
MRI: Magnetic resonance imaging
CEA: Carcinoembryonic antigen
AFP: Alpha-fetoprotein
DCP: Des-gamma-carboxy prothrombin
SMA: Smooth muscle actin
